# *LIN28A* polymorphisms and hepatoblastoma susceptibility in Chinese children

**DOI:** 10.7150/jca.52621

**Published:** 2021-01-01

**Authors:** Zhonghua Yang, Yuyao Deng, Keren Zhang, Yuzuo Bai, Jinhong Zhu, Jiao Zhang, Jiwen Cheng, Li Li, Jing He, Weilin Wang

**Affiliations:** 1 Department of Pediatric Surgery, Shengjing Hospital of China Medical University, Shenyang 110004, Liaoning, China.; 2 Department of Clinical Medicine, The Fourth Affiliated Hospital of China Medical University, Shenyang 110001, Liaoning, China.; 3 Department of Clinical Laboratory, Biobank, Harbin Medical University Cancer Hospital, Harbin 150040, Heilongjiang, China.; 4 Department of Pediatric Surgery, the First Affiliated Hospital of Zhengzhou University, Zhengzhou 450052, Henan, China.; 5 Department of Pediatric Surgery, the Second Affiliated Hospital of Xi'an Jiaotong University, Xi'an 710004, Shaanxi, China.; 6 Kunming Key Laboratory of Children Infection and Immunity, Yunnan Key Laboratory of Children's Major Disease Research, Yunnan Institute of Pediatrics Research, Yunnan Medical Center for Pediatric Diseases, Kunming Children's Hospital, Kunming 650228, Yunnan, China.; 7 Department of Pediatric Surgery, Guangzhou Institute of Pediatrics, Guangdong Provincial Key Laboratory of Research in Structural Birth Defect Disease, Guangzhou Women and Children's Medical Center, Guangzhou Medical University, Guangzhou 510623, Guangdong, China.

**Keywords:** Hepatoblastoma, Susceptibility, *LIN28A*, Polymorphism

## Abstract

Hepatoblastoma (HB) is the most prevalent primary hepatic cancer in children aged 6 months to 3 years. *LIN28A* is recurrently mutated in various diseases, and critically involved in tumorigenesis. However, a limited number of studies have examined the involvement of *LIN28A* polymorphisms in HB risk. We used the TaqMan assay to genotype four *LIN28A* polymorphisms (rs3811464 G>A, rs3811463 T>C, rs34787247 G>A, and rs11247957 G>A) in 275 Chinese children with HB and 1018 cancer-free controls from five medical centers in China. Their association with HB risk was evaluated on the basis of odds ratio (OR) and corresponding 95% confidence interval (CI). Overall, no significant associations were found in single locus and combine analysis. Interestingly, in the stratified analysis, we found that subjects with 1-3 risk genotypes were more likely to develop HB in patients ≥17 months of age (adjusted OR=1.76, 95% CI=1.04-2.98, *P*=0.034). The rs3811464 GA/AA genotypes were associated with decrease HB risk in patients with clinical stage III+IV disease (adjusted OR=0.50, 95% CI=0.26-0.96, *P*=0.038). Our results suggest that the *LIN28A* polymorphisms have a weak association with HB susceptibility in the Chinese children. *LIN28A* rs3811464 G>A may decrease HB risk in stage III+IV patients which need further validations with larger samples and different ethnicities.

## Introduction

Hepatoblastoma (HB) is the most prevalent primary hepatic cancer of embryonic origin 1. HB frequently exhibits a combination of histological patterns because of repeating stages in liver development 2. The incidence of HB peaks between the ages of 6 months and 3 years, and HB is more frequently seen in males 3. Over the last 2-3 decades, due to advances in multimodal treatments including surgical resection and adjuvant chemotherapy, 5-year overall survival rates have achieved as high as 80% 4. Meantime, as a result of low-birth-weight infants and the enhanced survival of premature babies, the incidence of HB appears to be gradually increasing, with about 4% per year for the period of 1992 to 2004 and a current rate of 1.5 cases/million 5-7. Furthermore, non-resectable tumors or metastases are associated with a poor prognosis 4, 8. To improve prognosis in HB, it is essential to comprehensively understand its pathogenesis 5, 9.

The most of HB is sporadic, but there are some familial cases associated with constitutional genetic abnormalities, such as familial adenomatous polyposis, trisomy 18 syndrome and Beckwith-Wiedemann syndrome (BWS) 10-12. Interestingly, single nucleotide polymorphisms (SNPs) have been found to be associated with the development and poor outcome of HB. Kim *et al*. used SNP array for whole-exome sequencing and analyses of genome-wide loss of heterozygosity and copy number in a BWS infant with HB. They found 11p15.5 uniparental disomy and *APC* and *PALB2* mutations at the germline level, and chromosome 1q gain and *CTNNB1* hotspot mutation at the somatic level 13. One study used high-density genome-wide SNP microarray analyses and demonstrated that 88% cases had chromosomal aberrations such as 1q,17q and 11q, among others 14. A previous study also demonstrated that* LINC00673* rs11655237 C>T polymorphism is significantly associated with HB risk and that rs11655237 T allele carriers are more likely to develop HB 15. These results suggested that complicated genomic abnormalities may contribute to the pathogenesis of HB.

LIN28 is an RNA-binding protein that was first shown to be a heterochronic gene controlling developmental timing. It is highly expressed during embryogenesis and plays a vital role in cell growth and embryonic development 16. Moreover, *LIN28* inhibits let-7 miRNA biogenesis and blocks pre-let-7 processing in the cytoplasm. It is known that the let-7 miRNA family targets oncogenic genes and downstream signaling pathways, thereby suppressing the progression of cell cycle and oncogenesis. Therefore, *LIN28* acts as an oncogene by inhibiting the synthesis of let-7 17. Mammals possess two *LIN28* homologs, namely *LIN28A* and *LIN28B* 18. *LIN28A* resides on chromosome 1p36.11, which blocks the biogenesis of all let-7s and promotes tumor growth 19. The temporal regulation of *LIN28A* expression at neurulation blocked axial elongation and embryonic growth in mice 20. The upregulation of *LIN28A* by Wnt could enhance the implantation potential of human embryo surrogate spheroids 21. Besides, Chang *et al*. identified the loss-of-function variant of *LIN28A* in patients with early-onset Parkinson's disease. They found that correction of the *LIN28A* variant in the donor patient's human induced pluripotent stem cells-improved behavioral phenotypes in the Parkinson's disease model 22.

Moreover, *LIN28A* (rs6598964 G>A) has been associated with increased mortality risk in colorectal cancer 23. In a hospital-based case-control study involving 1004 cases and 1296 controls, rs3811463 and rs6697410 in *LIN28* were found to be associated with breast cancer risk 24. However, the effect of the *LIN28A* polymorphisms on HB has not been studied. In the present study, we examined whether SNPs in *LIN28A* biogenesis genes confer HB risk in the Han Chinese population.

## Material and methods

### Patients and controls

In this study, we enrolled 275 cases with newly histopathologically diagnosed HB and 1018 non-cancer controls from five medical centers in Guangdong, Henan, Shaanxi, Yunnan and Liaoning provinces (**Table S1**). The 275 included children were examined and surgically treated in these five medical centers, diagnosed pathologically as HB, and finally staged, excluding HB cases that were examined only and did not undergo surgery, or underwent puncture biopsy only. Non-cancer controls were frequency-matched on age and sex were recruited from the same residing area as cases. Included patients provided their necessary written informed consent and trained interviewers collected their demographic information. The complete criterion for selecting participants was described previously 25, 26. Approval for this study was obtained from the ethics committee of each participating hospital. The study protocol was complied with ethical guidelines.

### SNP selection and genotyping

Four *LIN28A* polymorphisms (rs3811464 G>A, rs3811463 T>C, rs34787247 G>A, and rs11247957 G>A) were identified through the dbSNP database (http://www.ncbi.nlm.nih.gov/SNP) and SNPinfo online software (https://snpinfo.niehs.nih.gov/snpinfo/snpfunc.html) as we described previously 27, 28. Then the DNA samples were further genotyped using a standard commercial TaqMan real-time PCR kit 29. About 10% of the samples were randomly selected and re-genotyped for quality control. We obtained 100% agreement for the quality control samples.

### Statistical analysis

We used χ^2^ test to evaluate the difference in the distribution of characteristics, risk factor, and genotypes of* LIN28A* SNPs between cases and controls. The goodness-of-fit χ^2^ test was used to check if the frequency distributions of SNP genotypes were in accordance with Hardy-Weinberg equilibrium (HWE) in controls. We performed unconditional univariate and multivariate logistic regression analyses to determine the strength of the association between the selected polymorphisms and risk of HB through the odds ratios (ORs) and 95% confidence intervals (CIs). Adjustment for age and sex was performed in multivariate analysis. We also carried out stratification analysis based on age, sex, and clinical stages. *P*<0.05 was considered as statistically significant. All statistical analyses were two-sided and carried out using SAS software (version 9.1; SAS Institute, Cary, NC, USA).

## Results

### Relationship between *LIN28A* SNPs and HB susceptibility

Genotyping was successed in 275 HB cases and 1018 controls. All the four SNPs were in accordance with HWE in controls (rs3811464 G>A, *P*=0.165; rs3811463 T>C, *P*=0.823; rs34787247 G>A, *P*=0.193; and rs11247957 G>A, *P*=0.622). No significant association was found between *LIN28A* polymorphisms and HB susceptibility in either single locus or combined analysis (**Table 1**).

### Stratification analysis of association between *LIN28A* polymorphisms and HB risk

In the stratification analysis by age, sex and clinical stages (**Table 2**), we found significant association with the rs3811464 polymorphism (GA/AA vs. GG: adjusted OR=0.50, 95% CI=0.26-0.96, *P*=0.038) in patients with clinical stages III+IV. Regarding the combined analysis, we found that the 1-3 risk genotypes carriers were at significantly higher HB risk in patients ≥17 months of age (adjusted OR=1.76, 95% CI=1.04-2.98, *P*=0.034).

## Discussion

HB is the most prevalent primary hepatic cancer in children and accounts for around 1% of all childhood cancers 30, 31. Currently, the 5-year survival rate remains only 20-30% in cases with considerable unifocal and distant metastases at diagnosis 1, 32. A number of studies have reported that HB is related to genetic syndromes. For instance, 7.5-13.5% of children with BWS develop embryonal tumors, with the most frequent tumors being HB and nephroblastoma 31. Studies have also demonstrated that Wnt/β-catenin signaling pathway abnormalities exist in most cases of HB 33. Elevated expression of hTERT (human telomerase reverse transcriptase) and c-MYC appear to be predictive of a poor prognosis because both of them play crucial roles in the activation of Wnt/β-catenin signaling in aggressive phenotypes of HB 34, 35. With further study of HB pathogenicity genes, researchers have found that methylation and polymorphism of genes, microRNAs and lncRNAs are also related to the carcinogenesis of HB 15, 36-38. However, there has been no prior study about the association of *LIN28A* polymorphisms with HB susceptibility.

There have been many studies underscoring the importance of LIN28A in the regulation of stem cell pluripotency and self-renewal; however, recent investigations have shown abnormal expression of* LIN28A* in a broad array of tumor types, which is connected to advanced disease and poor prognosis 39. The findings of various studies have suggested that *LIN28A* gene polymorphisms play a role in cancer risk. Rao *et al*. found that LIN28A was a good immunohistochemistry marker for the diagnosis of embryonal tumor with multilayered rosettes 40. Li *et al*. showed that the downregulation of *LIN28A* expression suppressed differentiation and self-renewal of human breast cancer stem cells by inactivating the Wnt pathway in a let-7b-dependent manner 41. A case-control study at four centers demonstrated that *LIN28A* gene polymorphisms alter susceptibility to neuroblastoma; it was found that *LIN28A* SNPs, particularly rs34787247 G>A, could increase neuroblastoma risk 28. Also, *LIN28A* rs3811463 T>C and rs34787247 G>A have been associated with increased risk of Wilms tumor in Chinese children 27.

Our study showed that *LIN28A* polymorphisms affects HB susceptibility in a low-penetrating manner. We failed to find any single locos that can modify HB susceptibility. Stratified analysis indicated that rs3811464 GA/AA carriers had reduced risk to develop clinical stages III+IV tumors; the presence of 1-3 risk genotypes was significantly associated with HB risk among children ≥17 months of age. To our knowledge, we are the first group to confirm the association between *LIN28A* polymorphisms and HB susceptibility. Interestingly, we found that the presence of rs3811464 GA/AA genotypes in *LIN28A* provided a protective effect for children with clinical stages III+IV HB. Liu *et al*. studied the association between *APEX1* polymorphisms and neuroblastoma risk in Chinese children, and found that the rs1130409 GG genotype significantly reduced the tumor risk in males older than 18 months with 1-3 protective genotypes, and had a lower neuroblastoma risk 42. The effect of gene polymorphisms on cancer risk may be affected by tumor type, sample sizes, and selection criteria. Therefore, the association between *LIN28A* polymorphisms and HB risk should be specified in a particular population.

Our study demonstrated that *LIN28A* polymorphisms were not significantly associated with HB susceptibility. However, *LIN28A* rs34787247 G>A has been reported to be connected to increased risk of neuroblastoma and *LIN28A* rs3811463 T>C and rs34787247 G>A to increased risk of nephroblastoma 27, 28. Moreover, a study in East Asia showed that SNPs of *LIN28A* (rs11247954, rs12728900, rs3811463, rs4274112, rs4659441, rs6598964, and rs6683792) were not significantly associated with breast cancer risk 43. According to these findings, the effects of *LIN28A* polymorphisms on cancer susceptibility may be tumor type-specific. Single SNPs have been shown to be insufficient to modify tumor risk, while the occurrence of several SNPs could synergistically play significant roles in carcinogenesis 44.

Although this was the first and largest case-control study to assess the association between *LIN28A* polymorphisms and HB risk in the Chinese population to date, it cannot represent the entire Chinese population. Moreover, this study did not involve other races of people. Selection bias is an obvious possible confounding factor. Meantime due for phased research results of this study, there was no verification. We need to expand the sample size to further confirm the above results. To better determine the association of *LIN28A* polymorphisms with susceptibility to HB, further investigations should be designed to consider related factors including environmental background and data on parental diet, lifestyle, and exposure to hazards.

Overall, this study demonstrates that *LIN28A* SNPs have a weak relationship with HB susceptibility in the Chinese population.

## Supplementary Material

Supplementary table S1.

## Figures and Tables

**Table 1:** Association of *LIN28A* polymorphisms with hepatoblastoma susceptibility

Genotype	Cases (N=275)	Controls (N=1018)	*P*^ a^	Crude OR (95% CI)	*P*	Adjusted OR (95% CI) ^b^	*P*^ b^
**rs3811464 G>A (HWE=0.165)**							
GG	208 (75.64)	744 (73.08)		1.00		1.00	
GA	61 (22.18)	246 (24.17)		0.89 (0.64-1.22)	0.462	0.89 (0.64-1.22)	0.456
AA	6 (2.18)	28 (2.75)		0.77 (0.31-1.88)	0.560	0.77 (0.32-1.89)	0.573
Additive			0.367	0.88 (0.67-1.16)	0.367	0.88 (0.67-1.16)	0.368
Dominant	67 (24.36)	274 (26.92)	0.394	0.88 (0.64-1.19)	0.394	0.87 (0.64-1.19)	0.392
Recessive	269 (97.82)	990 (97.25)	0.601	0.79 (0.32-1.92)	0.602	0.80 (0.33-1.94)	0.616
**rs3811463 T>C (HWE=0.823)**							
TT	203 (73.82)	741 (72.79)		1.00		1.00	
TC	64 (23.27)	254 (24.95)		0.92 (0.67-1.26)	0.603	0.92 (0.67-1.26)	0.613
CC	8 (2.91)	23 (2.26)		1.27 (0.56-2.88)	0.568	1.28 (0.56-2.91)	0.553
Additive			0.912	0.99 (0.76-1.28)	0.913	0.99 (0.76-1.29)	0.931
Dominant	72 (26.18)	277 (27.21)	0.733	0.95 (0.70-1.28)	0.733	0.95 (0.70-1.29)	0.748
Recessive	267 (97.09)	995 (97.74)	0.532	1.30 (0.57-2.93)	0.533	1.31 (0.58-2.96)	0.520
**rs34787247 G>A (HWE=0.193)**							
GG	213 (77.45)	793 (77.90)		1.00		1.00	
GA	55 (20.00)	206 (20.24)		0.99 (0.71-1.39)	0.972	0.99 (0.71-1.38)	0.958
AA	7 (2.55)	19 (1.87)		1.37 (0.57-3.31)	0.481	1.38 (0.57-3.33)	0.474
Additive			0.727	1.05 (0.80-1.39)	0.727	1.05 (0.79-1.39)	0.733
Dominant	62 (22.55)	225 (22.10)	0.875	1.03 (0.75-1.41)	0.875	1.02 (0.74-1.41)	0.886
Recessive	268 (97.45)	999 (98.13)	0.477	1.37 (0.57-3.30)	0.478	1.38 (0.58-3.32)	0.470
**rs11247957 G>A (HWE=0.622)**							
GG	265 (96.36)	987 (96.95)		1.00		1.00	
GA	10 (3.64)	31 (3.05)		1.20 (0.58-2.48)	0.620	1.22 (0.59-2.52)	0.593
AA	0 (0.00)	0 (0.00)		/	/	/	/
Additive			0.620	1.20 (0.58-2.48)	0.620	1.22 (0.59-2.52)	0.593
Dominant	10 (3.64)	31 (3.05)	0.620	1.20 (0.58-2.48)	0.620	1.22 (0.59-2.52)	0.593
**Risk genotypes ^c^**							
0	55 (20.00)	239 (23.48)	0.175	1.00		1.00	
1	207 (75.27)	743 (72.99)		1.21 (0.87-1.69)	0.258	1.21 (0.87-1.69)	0.251
2	13 (4.73)	34 (3.34)		1.66 (0.82-3.36)	0.157	1.67 (0.83-3.37)	0.154
3	0 (0.00)	2 (0.20)		/	/	/	/
0	55 (20.00)	239 (23.48)		1.00		1.00	
1-3	220 (80.00)	779 (76.52)	0.222	1.23 (0.88-1.71)	0.223	1.23 (0.89-1.71)	0.216

OR: odds ratio; CI: confidence interval; HWE: Hardy-Weinberg equilibrium. ^a^ χ^2^ test for genotype distributions between hepatoblastoma patients and cancer-free controls. ^b^ Adjusted for age and gender. ^c^ Risk genotypes were rs3811464 GG, rs3811463 CC, rs34787247 AA, and rs11247957 GA.

**Table 2:** Stratification analysis of protective genotypes and hepatoblastoma susceptibility

Variables	rs3811464 (cases/controls)		AOR (95% CI) ^a^	*P* ^a^	rs3811463 (cases/controls)		AOR (95% CI) ^a^	*P* ^a^	rs34787247 (cases/controls)		AOR (95% CI) ^a^	*P* ^a^	Risk genotypes (cases/controls)		AOR (95% CI) ^a^	*P* ^a^
	GG	GA/AA			TT	TC/CC			GG	GA/AA			0	1-3		
**Age (month)**																
<17	108/336	40/123	1.01 (0.67-1.53)	0.960	107/339	41/120	1.08 (0.71-1.64)	0.710	117/357	31/102	0.93 (0.59-1.47)	0.757	36/107	112/352	0.95 (0.61-1.46)	0.804
≥17	100/408	27/151	0.73 (0.46-1.16)	0.185	96/402	31/157	0.83 (0.53-1.30)	0.420	96/436	31/123	1.15 (0.73-1.80)	0.557	19/132	108/427	**1.76 (1.04-2.98)**	**0.034**
**Gender**																
Females	84/296	29/104	0.97 (0.60-1.57)	0.908	79/302	34/98	1.32 (0.83-2.09)	0.242	87/308	26/92	1.00 (0.61-1.64)	0.991	24/94	89/306	1.15 (0.69-1.91)	0.594
Males	124/448	38/170	0.81 (0.54-1.21)	0.303	124/439	38/179	0.75 (0.50-1.13)	0.168	126/485	36/133	1.04 (0.69-1.58)	0.847	31/145	131/473	1.30 (0.84-2.00)	0.241
**Clinical stages**																
I+II	104/744	38/274	1.00 (0.67-1.48)	0.982	100/741	42/277	1.13 (0.77-1.67)	0.530	111/793	31/225	0.98 (0.64-1.50)	0.938	29/239	113/779	1.19 (0.77-1.84)	0.422
III+IV	60/744	11/274	**0.50 (0.26-0.96)**	**0.038**	58/741	13/277	0.60 (0.32-1.11)	0.106	53/793	18/225	1.20 (0.69-2.09)	0.522	11/239	60/779	1.67 (0.87-3.24)	0.126

AOR: adjusted odds ratio; CI: confidence interval. ^a^ Adjusted for age and gender, omitting the corresponding stratification factor.
