# Supplementary material for: LIN28A polymorphisms and hepatoblastoma susceptibility in Chinese children
Source: J Cancer. 2021 Jan 1;12(5):1373–8. doi: 10.7150/jca.52621 (PMC7847658; doi:10.7150/jca.52621)
Supplement: Supplementary file 1 — Supplementary table S1. [file jcav12p1373s1.pdf]

Table S1. Frequency distribution of selected variables in hepatoblastoma patients and cancer-free controls

Variables	Cases (N=275)		Controls (N=1018)		*P* [a]
	No.	%	No.	%	
Age range, month	0.10-149.97		0.033-156.00		0.365 [b]
Mean ± SD	23.81 ± 25.82		25.10 ± 19.35		
<17	148	53.82	459	45.09	
≥17	127	46.18	559	54.91	
Gender					0.589
Female	113	41.09	400	39.29	
Male	162	58.91	618	60.71	
Clinical stages					
I	93	33.82	/	/	
II	49	17.82	/	/	
III	51	18.55	/	/	
IV	20	7.27	/	/	
NA	62	22.55	/	/	

SD, standard deviation; NA, not available.

[a] Two-sided χ^2 test for distributions between hepatoblastoma patients and cancer-free controls.

[b] T-test for age distribution between hepatoblastoma patients and cancer-free controls.
